# What would encourage help-seeking for memory problems among UK-based South Asians? A qualitative study

**DOI:** 10.1136/bmjopen-2015-007990

**Published:** 2015-09-11

**Authors:** Naaheed Mukadam, Amy Waugh, Claudia Cooper, Gill Livingston

**Affiliations:** 1 Division of Psychiatry, University College London, London, UK; 2 Trainee Clinical Psychologist, University of Liverpool, Liverpool, UK

**Keywords:** QUALITATIVE RESEARCH

## Abstract

**Objectives:**

People from Minority Ethnic groups tend to present late to dementia services, often in crisis. Culture-specific barriers to help-seeking seem to underlie this. We sought to determine these barriers to timely help-seeking for dementia among people from South Asian backgrounds and what the features of an intervention to overcome them would be.

**Study design:**

Qualitative study to delineate barriers to and facilitators of help-seeking for South Asian adults with dementia through focus groups and individual interviews.

**Setting:**

Community settings in and around Greater London.

**Participants:**

To achieve a maximum variation sample, we purposively recruited 53 English or Bengali speaking South Asian adults without a known diagnosis of dementia through community centres and snowballing.

**Results:**

Participants ranged in age from 18 to 83 years, were mostly female and were 60% Bangladeshi. We recruited people from different religions and occupational backgrounds and included those with experience of caring for someone with dementia as well as those without this experience. Participants identified four main barriers to timely diagnosis: barriers to help-seeking for memory problems; the threshold for seeking help for memory problems; ways to overcome barriers to help-seeking; what features an educational resource should have.

**Conclusions:**

We have identified the features of an intervention with the potential to improve timely dementia diagnosis in South Asians. The next steps are to devise and test such an intervention.

**Strengths and limitations of this study**

- We purposively recruited a relatively large sample of South Asian people, giving a maximum variation sample.
- We specifically focused on exploring how to create an intervention to encourage more help-seeking within this ethnic group, which has not been done before.
- Our findings cannot be extrapolated to other underserved groups.

## Background

It is estimated that there are 850 000 people living with dementia in the UK, and the number of people affected is expected to increase to over one million by 2025.^1^ Black and Minority Ethnic (BME) people account for 15% of the English population and 39% of the London population.^2^ The largest minority group are of South Asian origin accounting for around 7% of the population in England and Wales.^3^ The BME population in the UK and other Western countries is younger than the majority population and tends to have a higher proportion of younger onset dementia,^4^ ^5^ so the overall burden of dementia is substantial.

People from BME backgrounds underuse services for dementia in the UK, USA and Australia.^6–8^ This inequality of service use is thought to lead to poorer dementia outcomes among BME groups and has led to UK policy of ensuring that services are culturally targeted and appropriate and suggesting creating special memory services for BME groups.^5^ There is debate about the benefits of earlier diagnosis of dementia, given the lack of curative treatments,^9^ and routine screening is unlikely to be of benefit,^10^ but earlier diagnosis enables people to make an informed choice about using services and future care, reduces family carer stress and delays institutionalisation. Equal access to dementia services for all ethnic groups is important to ensure these potential health benefits. In the UK, as in other Western countries, early diagnosis of dementia is a national priority.^11^

We reported in a systematic review that barriers to accessing dementia services described in a broad range of BME groups include: attributing the symptoms to normal ageing or other physical, spiritual or psychological causes; denial that there was a problem or normalisation of symptoms; concerns about stigma related to dementia; perceived ethical imperative to care for one’s own family members without accessing help; negative experiences of the healthcare service and feeling there was nothing that could be done for dementia. The only facilitator to help-seeking found was knowledge about dementia.^12^ In order to reduce healthcare inequalities, it is important that diverse needs are identified of differing BME groups.^13^ Only two of the studies reviewed directly compared help-seeking for dementia in different ethnic groups.

Our previous small study comparing BME groups with the white UK-born population found that certain barriers to help-seeking seemed specific to BME groups, such as different beliefs about the aetiology of symptoms, concerns about stigma and the perceived benefit of looking after your own family until you could no longer cope. Diagnosis alone was also less valued among BME carers than their white UK counterparts.^14^ A systematic review specifically focused on dementia and mental illness in people of South Asian origin also found stigma, lack of knowledge about services and culturally preferred coping strategies to be significant barriers to help-seeking.^15^

Thus, barriers to help-seeking for dementia are culturally specific and encouraging help-seeking in BME groups should take these particular concerns into account. Simply providing information about an illness is not sufficient to change health-related behaviour,^16^ so we sought to explore what factors would encourage help-seeking among UK-based South Asians.

## Aims

We aimed to explore, in a larger population, South Asian peoples’ interpretation of cognitive symptoms and the reluctance behind seeking medical help for these symptoms. We also wanted to explore for the first time what might encourage earlier help-seeking in order to devise an intervention to encourage timely diagnosis.

## Methods

### Participants

We defined South Asian as being anyone who identified themselves as having South Asian identity or heritage by links to any South Asian country, primarily India, Pakistan, Bangladesh, Nepal and Sri Lanka. We purposively recruited participants from South Asian community centres and then through snowballing from those contacts and researchers’ personal or professional contacts, in order to gain a maximum variation sample and therefore range of opinions; aiming for participants from either gender, a range of marital status, ages, educational background and occupations; people born in the UK and a variety of South Asian countries, and from differing religions. We recruited participants who wished to be in a single-sex only group and those who were comfortable in a mixed sex group and included participants with and without experience of caring for or interacting with people with dementia.

### Procedures

All participants provided written consent after reading a participant information sheet and having the opportunity to ask questions. This was done by trained researchers using the principles of the Mental Capacity Act (http://www.legislation.gov.uk/ukpga/2005/9/contents). We conducted focus groups in English or Bengali with an interpreter as needed. Participants were included if they spoke other South Asian languages but could participate in English, but Bengali speakers did not need to be able to speak English. Bengali was chosen as it is one of the most commonly spoken South Asian languages in the UK.^17^

We organised focus groups so they were comprised of people with shared background, for example, attending the same community centre as from the same country of origin, as homogeneity within the group can facilitate more detailed and free flowing consideration of topics and also to allow comparison between groups.^18^ ^19^ They were also used to allow group processes to enable participants to explore and clarify their views in ways that may not have been possible using individual interviews.^19^ We conducted individual interviews with some participants(eg, a family carer) to explore their experiences in more detail and in a more private setting,^20^ and in some cases these were conducted if there were only one or two participants at a centre. We collected demographic information for all participants. Those who were willing and able also answered some written questions about dementia aetiology, attitude to help-seeking for dementia and their own experiences in caring for someone with dementia. Interviewees were given £20 in tokens to thank them for their time.

The interview began with reading out a case vignette describing a South Asian woman with significant memory problems. This was developed in order to describe a person with significant memory problems justifying further investigation, and to ensure content validity we modified it after consultation with memory clinic doctors. Participants were asked what they thought her symptoms were due to and how she should deal with them. Where interpreters were used, they translated the vignette verbatim and spoke it aloud, then translated all facilitator comments and questions as they were issued. Discussion about barriers to help-seeking and how to encourage help-seeking for these kinds of problems was then encouraged using prompts based on previously identified barriers to help-seeking. The interview guide was continually modified according to emerging concepts from focus groups and interviews. Recruitment continued until theoretical saturation was achieved, that is, no new themes were emerging from the data.^21^

### Data analysis

Focus groups and individual interviews were audiotaped and transcribed verbatim. Transcripts of individual interviews were sent to participants for validation and comment. Two researchers (NM and AW) analysed the transcripts independently using interpretative phenomenological analysis.^22^ This method was selected because it is best suited to exploring the meaning and significance of experiences of participants in order to gain insight into psychosocial processes. Inductive rather than deductive analysis was applied as there were no specified hypotheses to test and we wished to build a knowledge base up from observations, as is common practice in qualitative research.^23^ Emerging themes were discussed and compared across demographic categories. The codes and themes were refined in an iterative process and a final coding scheme was agreed by consensus and with discussion with a third researcher (GL) where needed. We used NVIVO software (QSR International Pty Ltd, V.9, 2010) for our analysis. We have anonymised all quotations, providing non-specific demographic information.

## Results

### Participants and demographics

We conducted seven focus groups and five individual interviews, with 53 participants in total. The focus groups were held in and around London in a Bengali women's group, a Bengali men's group, an Ismaili community centre, a Hindu cultural group and an Asian women's group. Individual interviews were conducted on University College London premises, in community centres or participants’ workplaces. Participants were 58.5% female with a mean age of 57 (range 18–83 years). Most (74%) identified themselves as Muslim and 60% were of Bangladeshi origin. Their demographic characteristics are shown in table 1.

**Table 1:** Demographic characteristics of participants

Characteristic	Number (percentage)
Mean age (range)	57 (18–83)
Female	31 (58.5)
Ethnicity:	
Bangladeshi	32 (60.4)
Indian	17 (32.1)
Pakistani	2 (3.8)
Other	2 (3.8)
Place of birth	
Bangladesh	31 (58.5)
Africa (various countries)	12 (22.7)
India	6 (11.3)
Pakistan	1 (1.9)
UK	2 (3.8)
Other	1 (1.9)
Mean years in UK (range)	32.0 (4–51)
First language	
Bengali	30 (56.6)
Gujarati	7 (13.2)
English	8 (15.1)
Hindi/Urdu	5 (9.4)
Punjabi	3 (5.7)
Marital status	
Married/living with partner	35 (66.0)
Single	6 (11.3)
Widowed	6 (11.3)
Separated	1 (1.9)
Unknown	5 (9.4)
Religion	
Islam	39 (73.6)
Hinduism	10 (18.9)
Jain	1 (1.9)
Christian	1 (1.9)
Agnostic	1 (1.9)
Unknown	1 (1.9)
Age at leaving full-time education (50 participants)	
No formal education	7 (14.0)
<10	2 (4.0)
10–18	23 (46.0)
19–25	10 (20.0)
>25	6 (12.0)
Unknown	2 (4.0)
Employment:	
Never worked	13 (24.5)
Current/previous occupation	37 (69.8)
Full-time education	3 (5.7)
Unknown	2 (3.7)
SOC Group (2010)	
1. Managers, directors and senior officials	2 (3.7)
2. Professional occupations	8 (15.0)
3. Associate professional and technical occupations	1 (1.9)
4. Administrative and secretarial occupations	6 (11.3)
5. Skilled trades and occupations	6 (11.3)
6. Caring, leisure and other service occupations	3 (5.6)
7. Sales and customer service occupations	2 (3.7)
8. Process, plant and machine operatives	1 (1.9)
9. Elementary occupations	6 (11.3)

SOC, Standard Occupational Classification.

Thirteen of 17 participants who filled in the more detailed questionnaire knew someone with dementia, four of whom had cared for someone who had dementia.

### Themes

We looked for patterns in the emergence of themes with regard to whether demographic characteristics or experience with dementia affected people's opinions but found no themes or codes that were exclusive to a particular group or participant characteristic. All themes are therefore presented as relating to the entire sample.

There were four main themes identified. These were: barriers to help-seeking for memory problems; the threshold for seeking help for memory problems; ways to overcome barriers to help-seeking and what features an educational resource should have. These are discussed below.

### Barriers to help-seeking

We divided the barriers discussed into those that occur at the individual level, the societal/community level and the healthcare system level. This grouping was based on the main themes that emerged in group discussions and mapped onto previously described barriers to help-seeking as well as to the theoretical framework described in the Theory of Planned Behaviour which can inform future interventions.^24^ These are shown in table 2, along with how frequently they were mentioned in total across all groups and interviews.

**Table 2:** Barriers to help-seeking for memory problems

	Frequency
**Individual**	
*Lack of acknowledgement that there is a problem due to:*	
Fear of institutionalisation	5
Lack of language to describe problems	4
Denial from individual	3
Fear of the diagnosis itself	3
Unwillingness to challenge family hierarchy	2
Wish to maintain position in society	1
Lack of communication	2
*Believing that memory problems are due to:*	
Old age	9
Social isolation/stressors	9
Psychological cause/mental illness	6
Another physical illness	2
Spiritual cause	1
*Feeling that responsibility for getting better lies with:*	
The individual themselves	2
The family	1
God	1
**Societal**	
Perceived stigma of mental illness	10
Stigma of cognitive symptoms	6
Expectation that family should look after their own as long as possible	4
Feeling that dementia is a dangerous illness	2
**Healthcare system**	
Not knowing what help is available	10
Feeling nothing can be done	4
Language barrier	3
Lack of culturally appropriate help	2
Feeling that the diagnosis itself can never be certain	1
*Perception that general practitioners:*	
Do not have enough time in consultations	5
Are not useful as a first point of contact	3
Would not take concerns seriously	3
Would say that memory problems are due to old age	2

#### Individual-level barriers to help-seeking

##### Memory problems are an inevitable and normal part of ageing

Some participants in each focus group commented that they frequently witnessed memory problems among older members of their families and communities or even among themselves. They regarded these as inevitable and normalised their occurrence, even where their descriptions suggested more serious problems.

In reality, most of them are having the same problem. At least so many times in a day, my mum, that aunty, they forget things where they put it. Participant number 1, 60–69yo Bangladeshi Muslim woman, no education, focus group

Something is attributed just to old age and it's going to come and there's nothing you can do about it. P2, 50–59yo Indian Jain woman, tertiary education, interview

#### Memory problems are not an illness

Another common belief was that social isolation or stressors could cause symptoms.

The children have grown up, they have their own life, they are not living with parents, and they are feeling lonely, isolated. That's another problem for memory problems. P3, 60–69yo Bangladeshi Muslim woman, minimal education, focus group

The idea that memory problems may be due to spiritual wrongdoing was mentioned.

It's just that you may not have done something in your previous life, you know, that you're getting some of these problems. P2, 50–59yo Indian Jain woman, tertiary education, interview

One participant expressed the view that it would be up to God to decide if the person has an illness for which help should be sought, although no explanation was given for how this would guide the decision-making process.

Allah knows if it is an illness or not. P4, 60–69yo Bangladeshi Muslim female, no education, focus group

#### Individuals or families can make memory problems better

Other participants said that the person or their family could make themselves better.

So he needs to share his pain or sorrow with a friend who can be assisting, can be supporting him or her. The more you share, you get more breathing space, so you can think better. The dementia we can cure or we build ourself…We need to come out of these things somehow. P5, 50–59yo Bangladeshi Muslim man, tertiary education, focus group

#### Societal-level barriers to help-seeking

##### Stigma of diagnosis

Another common theme was the stigma of mental illness and cognitive decline, which is classed as a type of mental illness.

There is a lot of stigma attached to psychiatric problems and memory falls within that domain. P6, 25–29yo Pakistani agnostic man, post-graduate student, interview

Some participants in each group thought that the stigma of a memory disorder was much worse than that of a chronic medical disorder.

Most illnesses,…you can be distanced from them and you can deal with them on a practical level. You get diagnosed. You're treated, you do something to make it go away, or live with it, but there is a sense of separation, but with dementia it's more devastating, I think, because it completely takes over the person…it actually takes you away from who you are as a human being, with all these connections that we have. P7, 50–59yo Indian Muslim man, secondary education, interview

Several participants agreed that the stigma in the UK would be less than in their home country.

The stigma is less here than it would be back home. Back home our families would be sort of outcast, people would avoid going to their homes if they knew there was somebody suffering with this kind of problem. P8, 50–59yo Indian Muslim woman, secondary education, focus group

Participants also linked the stigma of dementia to ideas about dangerousness from neglect.

People who are…have dementia not only mentally but sometimes become incontinent or they might be a danger to themselves or the society; they might walk away from the house or set the burner on or something. P9, 70–79yo Indian Hindu man, tertiary education, focus group

#### Good families look after people with dementia themselves

There was a perceived expectation in the community that families would provide care when relatives experience memory problems, without outside help. This was mentioned by a few participants and linked with respect for the affected person and the family hierarchy.

It is in a way seen as a badge of pride if the family is looking after them whereas there is a lot of stigma in being transferred to mental health services or a care home where they could probably be better looked after. P6, 25–29yo Pakistani agnostic man, post-graduate student, interview

You couldn't quite discuss that he had memory problems with the wider family or whatever. It's just not done, that would be disrespectful so you know you cope. P2, 52yo Indian Jain woman, tertiary education, interview

One participant commented that families might only feel able to ask for help when the person with memory problems was near the end of their lives.

You deal with it as best as you can. Maybe they start thinking about receiving help in terms of terminal care. P10, 25–29yo Indian Hindu woman, post-graduate student, interview

Some expressed the view that there was more support available in their home country but this was not accepted by all participants.

Back home we've got a lot of support in these sort of things. But here, you are stuck in the world by yourself. P5, 50–59yo Bangladeshi Muslim man, tertiary education, focus group

I don't think so, it's same as here…There are worries everywhere, any country, I believe. P11, 60–69yo Fijian Muslim woman, secondary education, focus group

#### Healthcare system-level barriers to help-seeking

##### Lack of knowledge of help available

A commonly mentioned barrier was being unaware of what services were available for cognitive problems.

People don't know about the services….there's lack of knowledge about the services. P6, 25–29yo Pakistani agnostic man, post-graduate student, interview

Many participants said that they would see their general practitioner (GP) in the first instance about memory problems and expressed the view that health and social care support was very good in the UK. Others had reservations, saying that GP time was limited and GPs would prioritise severe dementia and physical illnesses and dismiss memory problems as being due to old age. Some participants also felt that services needed to have a better understanding of cultural needs and the lack of it led to worse outcomes.

There are some multifactorial issues as to why Asians don't seek (help) and when they do seek then there isn't a lot of information provided to them so it prevents them from seeking help in the first place and if they do end up in care homes…they aren't looked after as well as they could be. P6, 25–29yo Pakistani agnostic man, post-graduate student, interview

Some participants questioned the meaning of and certainty about a diagnosis.

Dementia, how certain are you, from after your diagnosis, that it is dementia, and it's not just general forgetfulness? P12, 40–49yo Indian Muslim woman, secondary education, focus group

One participant commented that memory and cognitive problems were not discussed at GP appointments until there was a crisis:

When they go to see the GP, the conversation of tell me what's happening to you, they come out with physical symptoms rather than you know impairment of other kinds…It doesn't come up until you're into crisis. P2, 50–59yo Indian Jain woman, tertiary education, interview

Some participants also mentioned that services may be harmful, feeling afraid that disclosing cognitive symptoms could result in having to leave their own home.

I think some people don’t want to tell other people that they’re forgetting, they're scared that they would think that they’re crazy or if they’re living alone, try and get them admitted or something. People are scared of that, you know. P8, 50–59yo Indian Muslim woman, secondary education, focus group.

### The threshold for help-seeking

Several participants in each group felt that help should be sought for memory problems as soon as possible. However, while many reported memory problems, only one had sought help.

Some participants said that if symptoms were more frequent or were troubling the individual, then they should ask for help, but this was difficult to quantify.

I think if it’s happening more often then it’s a cause for concern. P13, 60–69yo Indian Muslim woman, secondary education, focus group

More commonly, people said they would get help if there was a specific event that was severe or risky

If I forget an appointment, or if I forget the timing of an appointment, I'd consider that a minor matter and it wouldn't alarm me. But if I got up and I didn't know what day it was, or what the date was, or if I found myself and didn't know where I was or how I'd got there, then I think it would be a cause for me to be alarmed. P14, 40–49yo Pakistani Muslim woman, secondary education, interview

Participants also said that they would get help for behavioural changes, self-neglect, psychological symptoms like anxiety and any sort of risk, such as fire risk or not remembering if they had taken their medications. However, even the risk of fire was felt not to be significant unless it happened repeatedly.

There would be stronger signs if it's constant. If it's a one-off example you can bypass that. If it's constant, silly things like leaving the gas on, leaving the fire on and then it's getting more and more severe, then leaving the gas on is a very good danger sign for a family…when families need to be alerted, and say “ok, we've got a problem”. P15, 30–39yo Bangladeshi Muslim male, tertiary education, focus group

We have people that when they can't take care of themselves but if they have the capacity for the basics like helping with cooking or going for their walks or just taking care of their hygiene and if that is compromised then that is when we seek care but until then it didn't seem like it was necessary as it wasn't that severe. P10, 25–29yo Indian Hindu woman, post-graduate student, interview

### Ways to overcome barriers to help-seeking

We asked participants what would encourage them to seek help earlier for cognitive problems.

#### Normalising help-seeking and breaking down stigma

Several suggested that normalising help-seeking and breaking down the stigma associated with both the symptoms and help-seeking might encourage earlier help-seeking.

The only thing one can do is to tell the particular person that this is nothing uncommon and nothing to be embarrassed about. It happens to all of us and encourage her to seek medical help. P9, 70–79yo Indian Hindu man, tertiary education, focus group

#### Emphasis that dementia has a physical cause

Some participants mentioned other illness awareness campaigns that they felt had made a significant impact and this highlighted the importance of emphasising the physical rather than mental nature of illness.

If people were introduced to the fact that there is a physical cause…I think if things are explained in a simple and logical manner it'll become more approachable, and understandable, and acceptable. P14, 40–49yo Pakistani Muslim woman, secondary education, interview

Other ways of reducing reluctance to seek help were also used to consider the design of an intervention.

#### Use a trusted source

Several participants stated that the information should come from a trusted source. Letters from the National Health Service (NHS) and particularly from GPs were felt to have considerable impact and were likely to be read.

You see, because I find if any medical letter comes in, NHS, recently I got a couple of them so I’ll read through it whether it concerns me or not, I think one should know. P16, 70–79yo Indian Hindu man, tertiary education, focus group

#### Target the audience

Some participants felt information should be targeted at South Asian people of all ages, others suggested targeting older people, or those aged 30–40 or specifically women because they would be caring for older relatives. Most agreed that the literature should feature South Asian people:

Something you can identify with, because usually you know whatever you see, you see a grey haired person and usually a white person so you think well there isn't a connection. P2, 50–59yo Indian Jain woman, tertiary education, interview

#### Reducing barriers because of language and literacy

Participants suggested that information could be presented in leaflets, a DVD, TV adverts, and videos on GP practice waiting room screens or a combination of these. DVD was the format most frequently endorsed as this would provide more detailed information and use both English and south Asian languages.

Make it clear that the usefulness applies to south Asians by presenting it as a personal story.

I think the fact that you actually had a description of a person going through the problem caught my interest…That's how I relate to things. P14, 40–49yo Pakistani Muslim woman, secondary education, interview

#### Important information to include

Participants felt it was important to include information about symptom progression and when to seek help as many people might equate the term dementia with very severe dementia.

Understanding that you can carry on you know and just understanding the progression because usually people associate dementia with those last stage, you can't recognise anybody, you're incontinent—they only recognise that very end stage they don't recognise from the initial from the very start, that is not people's conception. P2, 50–59yo Indian Jain woman, tertiary education, interview

They felt the benefits of help-seeking should be emphasised.

While there isn't a cure, I think it's important to realise that there is a lot which can be done for the person during the process. P14, 40–49yo Pakistani Muslim woman, secondary education, interview.

## Discussion

This study is the first to consider in more detail the reasons behind the barriers to help-seeking for dementia in UK-based South Asians and what intervention might make a difference. We found as others did that there was stigma but more details of what it was about. Stigma was linked with ideas of “madness”, lack of physical aetiology and lack of treatment. Using a case discussion also helped to highlight the dilemma that people with cognitive problems are likely to face—namely, that forgetfulness is a common and normal experience, so it is difficult to differentiate it from significant cognitive impairment, but the study adds to the literature by finding a particularly high threshold for identifying abnormality in this group. In addition, we recruited a wider range of participants than previous studies including those with direct experience of caring for someone with dementia and by seeking potential solutions to the barriers found.

Overall, participants recognised memory problems and felt concerned about them, but there were many barriers to help-seeking, particularly the belief that memory decline was inevitable and not an illness. Reasons behind denial of symptoms, such as wanting to maintain one’s position in society or in the family hierarchy and the fear of institutionalisation and the stigma it carries, were interesting and useful. Thus, an intervention could tackle stigma around dementia by reframing it as a possible sign of an underlying physical illness. The study suggests that an intervention should give information about key symptoms which would lead to help-seeking and how that could be beneficial to the person with dementia, as well as the family. Thus, getting help is not seen as relinquishing one’’ responsibilities, but rather living up to them so that the person with dementia can live as fulfilling a life as possible. Equally, help-seeking could be useful in allaying the fears of those who are not developing dementia.

In this participant group, visual presentation of information was felt to be desirable and people felt more able to relate to a person's story rather than pure clinical information. The source of the information should be trustworthy and the NHS fell into this category. These preferences, while only subtly different to the views of people from other cultural or ethnic groups, represent what this group of South Asian participants felt was important and are the best representation of the views of UK-based South Asians to date; therefore, they are of value in developing a culturally relevant intervention. Some of these barriers to presentation may not be culturally specific, but an intervention which excluded general barriers would clearly not be likely to be effective.

### Strengths

This was a relatively large qualitative study with good variation in participant demographic characteristics. Sampling was purposive for characteristics which could have an impact on attitudes to help-seeking for dementia and continued until theoretical saturation was reached. Data analysis was iterative and carried out independently by two researchers to maximise the yield of themes and concepts.

The use of a case vignette and group discussions gave detailed and varied accounts of the help-seeking process. Individual interviews, particularly with professionals with experience of working with people with dementia, provided interesting insights based on personal experiences of working within the South Asian community. Overlap with previously identified barriers to help-seeking in dementia in minority ethnic groups suggests transferability of the findings (12;14).

### Limitations

While this relatively large qualitative study gave rich in-depth information about the views of 53 community members, it is not necessarily representative of that whole community, particularly as all participants were from around London and only spoke English or Bengali. South Asians are a large and heterogeneous group, but even in homogeneous groups there will be differences among individuals. There was, however, no apparent influence on opinions expressed according to demographic features, although we considered this in our analysis. Also, as we were asking people about a hypothetical case, it may be that their opinions would change should they face the same situation in reality.

## Conclusions

This study has further explored barriers to help-seeking for dementia in the South Asian community and highlighted ways to overcome these barriers. An intervention should be targeted at known barriers to help-seeking within this community, should use personal narratives and should come from a reliable and trusted source. This might improve help-seeking for dementia in this vulnerable community. We intend to go on to produce, refine and test such an intervention.
